# TGF-*β*1 of no avail as prognostic marker in lyme disease

**DOI:** 10.7717/peerj.398

**Published:** 2014-05-27

**Authors:** Julia Schumann

**Affiliations:** Center for Biotechnology and Biomedicine (BBZ), Faculty of Veterinary Medicine, University of Leipzig, Leipzig, Germany

**Keywords:** Inflammatory lessions, Lyme disease, TGF-β1, Prognostic marker

## Abstract

**Background.** Within the present *in vivo* study using the wild type mouse strains C3H/HeN and FVB/N it was intended to (1) measure TGF-*β*1 expression in the course of lyme disease, (2) examine the potential correlation of TGF-*β*1 expression with the clinical outcome of a *Borrelia* infection (with a focus on lyme arthritis), (3) develop a diagnostic tool based on the endogenous factor TGF-*β*1 to predict the progressivity of lyme disease.

**Findings.** In the course of lyme disease there was an increase in the serum content of active TGF-*β*1, which became significant 56 days post infection (*p* < 0.001). The serum concentration of total TGF-*β*1 in the course of infection initially decreased then rebounded and subsequently dropped again. Despite considerable individual variations in active TGF-*β*1 serum concentrations there were no identifiable dissimilarities in the clinical appearance of the mice. Likewise, no correlation could be seen between the serum content of active TGF-*β*1 and the severity of lyme arthritis of tibiotarsal joints of infected mice.

**Conclusions.** The present study clearly shows that TGF-*β*1 is of no avail as prognostic marker in lyme disease. Hence, the search for an endogenous predictive factor, which can be determined in an easy and reliable manner, remains open.

## Introduction

Lyme disease is a chronic, inflammatory condition which occurs frequently in humans and domestic animals (Steere, 2001). The causative agents are spiral bacteria species from the genus *Borrelia*. The bacteria are transmitted during the blood meal of hard-shelled ticks, actively migrate through the body, and establish a persistent infection (Steere, 2001). Months or years after infection the pathogens can still be isolated from the host (Straubinger et al., 2000). Organs typically affected include the joints, the heart and the nervous system. Presence of the spirochetes in these organ systems leads to chronic inflammations, namely arthritis, pericarditis and meningitis (Steere, 2001). Due to the permanent inflammatory response patients suffer from a marked loss of their quality of life. In the acute phase of the infection antibiotic treatment usually yields good results (Steere, 2001; Straubinger et al., 2000). However, a persistent infection mostly cannot be treated adequately using antibiotics (Straubinger et al., 2000). An early diagnosis regarding establishment and progression of a chronic disease process, therefore, would be highly beneficial for primary and follow-up therapies.

TGF-*β*1 is a central immunmodulatory cytokine which controls the activity of numerous immune cells. It is synthesized as a latent precursor molecule which is activated in a multistep process (Fortunel, Hatzfeld & Hatzfeld, 2000). The pleiotropic factor has complex biological actions which depend on the cell type as well as the developmental and the activation status of the target cell (Wahl, 1999). TGF-*β*1 is known to be of importance for a multitude of chronic inflammatory diseases such as autoimmune hepatitis, inflammatory bowel disease and rheumatoid arthritis thereby mediating detrimental and beneficial effects simultaneously contingent on the cell type influenced (Wahl, 1992; Wahl, 1994).

Development and severity of lyme arthritis has been shown to depend on a Th17 cell-mediated immune response (Burchill et al., 2003; Nardelli et al., 2004; Nardelli et al., 2008). The predominant cytokine synthesized and released by Th17 cells is IL-17. Treatment of *Borrelia* infected mice with anti-IL-17 antibodies is reported to inhibit the development of lyme arthritis (Burchill et al., 2003; Nardelli et al., 2004; Nardelli et al., 2008). In this regard it is important to note that TGF-*β*1 together with IL-6 drives the establishment of the IL-17 producing Th17 cells (Bettelli et al., 2006; Veldhoen et al., 2006). Consequently, simultaneous administration of anti-TGF-*β*1 and anti-IL6 antibodies to *Borrelia*-vaccinated and challenged mice resulted in an inhibition of lyme arthritis development as well (Nardelli et al., 2008).

The essential role of TGF-*β*1 in the development of lyme arthritis raised the question whether the cytokine may be of use as a prognostic marker of lyme disease. In fact, for hepatitis c, which as lyme disease is a chronic inflammatory condition, a correlation between the TGF-*β*1 content in blood serum of human patients and progression of liver fibrosis has already been described (Cecere et al., 2004). Thus, the goal of the present study was to elucidate the suitability of TGF-*β*1 as a predictive marker of the course of lyme arthritis.

Within the present *in vivo* study using the wild type mouse strains C3H/HeN and FVB/N it was intended to (1) measure TGF-*β*1 expression in the course of lyme disease, (2) examine the potential correlation of TGF-*β*1 expression with the clinical outcome of a *Borrelia* infection (with a focus on lyme arthritis), (3) develop a diagnostic tool based on the endogenous factor TGF-*β*1 to predict the progressivity of lyme disease.

## Materials and Methods

### Mice

For investigations female mice between 8 and 10 weeks of age of the wild type mouse strains C3H/HeN and FVB/N (Charles River Laboratories International Inc., Wilmington, USA) were used. Mice were maintained in an individually ventilated caging system under environmental conditions of 21°C, 50–60% humidity, 20 air changes/h and a 12:12 h light:dark cycle in accordance with the guidelines approved by the Animal Care and Usage Committee of the Regierungspräsidium Leipzig. Sterile food and water were given *ad libitum*. The mice were tested periodically for pathogens according to the recommendations for health monitoring of mice provided by the Federation of European Laboratory Animal Science Associations accreditation board. The experimental design was approved by the Leipzig Regional Council (Regierungspräsidium Leipzig), protocol number 24-9168.11-TVV13/05.

### Experimental design

Trials were conducted in groups of *n* = 7 for each time point investigated. In a first experiment the same mice were bled retro-orbitally multiple times during the course of the infection (0, 7, 14, 28 and 56 days post infection). In a second experiment different mice were sacrificed at every time point (0, 7, 14, 28 and 56 days post infection) to take cardiac blood samples together with the tibiotarsal joints of the animals.

### Cultivation of *Borrelia burgdorferi sensu stricto* N40 and infection of mice

*B. burgdorferi sensu stricto* N40 were grown for ten days at 33°C in modified Barbour–Stoenner–Kelly (BSK) medium as previously described (Knauer et al., 2007). Organisms were visualized by dark field microscopy and enumerated by using a Petroff-Hausser counting chamber. Anesthetized mice were infected using *B. burgdorferi sensu stricto* N40, passage 4, via injection of 100 µl BSK medium containing 1 × 10^7^ viable spirochetes intradermally into the shaved back.

### ELISA

Concentrations of TGF-*β*1 in the sera of mice 0, 7, 14, 28 and 56 days post infection were determined using the ELISA kit DuoSet^®^ ELISA Development System mouse TGF-*β* from R & D Systems (Wiesbaden, Germany) according to the manufacturer’s instructions. Both the free TGF-*β*1 content and the total TGF-*β*1 content, that is the content of active plus latent TGF-*β*1, were analyzed. For total TGF-*β*1 determination samples were preincubated using 2.5 N acetic acid and 10 M urea for 10 min and neutralized using 2.7 N sodium hydroxide and 1 M Hepes before ELISA analysis as recommended by the manufacturer.

### Histologic analysis

Tibiotarsal joints were removed from euthanized mice 0, 7, 14, 28 and 56 days post infection, fixed in 10% buffered formalin (VWR, Darmstadt, Germany), trimmed, embedded, cut and stained with hematoxylin and eosin (HE staining, VWR, Darmstadt, Germany) by standard procedures. Tissue slices were examined by pathologists in a blinded manner. The pathologic changes were classified using a histological score ranging from 1 to 6 with score 1 denoting no changes and score 6 denoting high-grade changes as previously described (Schumann & Blessing, 2011).

### Statistical analysis

Statistical analysis was carried out using the program GraphPad Prism 4 (GraphPad Software, La Jolla, USA). One-way analysis of variance followed by unpaired Students *t* test as well as linear regression analysis was used to identify significant differences and correlations between means. In all cases, *p* < 0.05 was assumed to indicate significance.

## Results and Discussion

### TGF-*β*1 expression due to *Borrelia* infection

**Figure 1:**
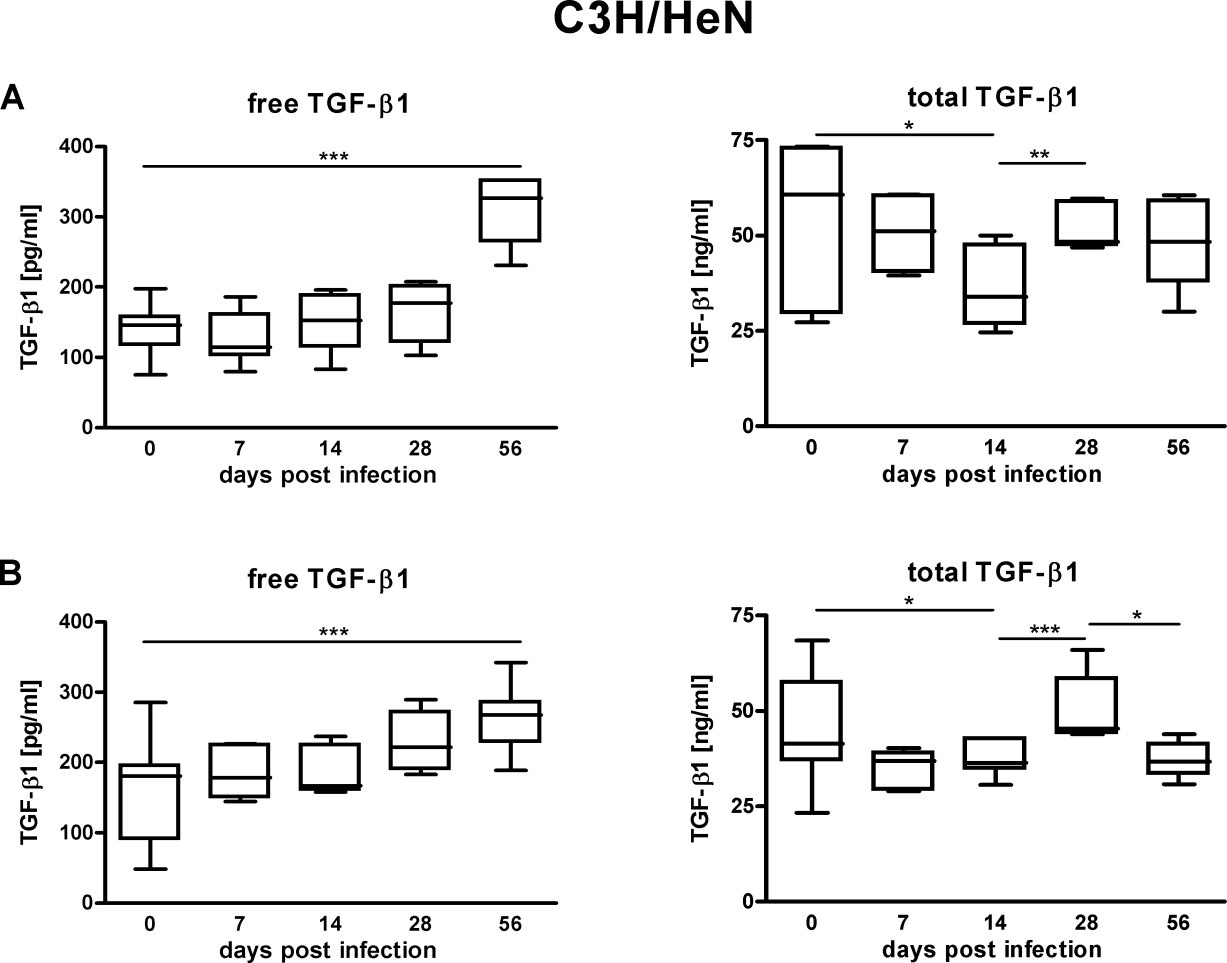
TGF-*β*1 content in the sera of C3H/HeN mice suffering from lyme disease. Measured concentrations of free (pg/ml) as well as total (ng/ml) TGF-*β*1 in the sera of C3H/HeN wild type mice 0, 7, 14, 28 and 56 days post infection with *B. burgdorferi sensu stricto* N40. Data are from 2 independent experiments with 7 animals tested at each time point (*N* = 2, *n* = 7). (A) Different mice were sacrificed at every time point to take cardiac blood samples. (B) The same mice were bled retro-orbitally multiple times during the course of the infection. Statistical analysis was carried out for each experiment using the program GraphPad Prism 4 (GraphPad Software, La Jolla, USA). One-way analysis of variance followed by unpaired Students *t* test was used to identify significant differences and correlations between means (* *p* < 0.05, **p < 0.01, *** *p* < 0.001).

**Figure 2:**
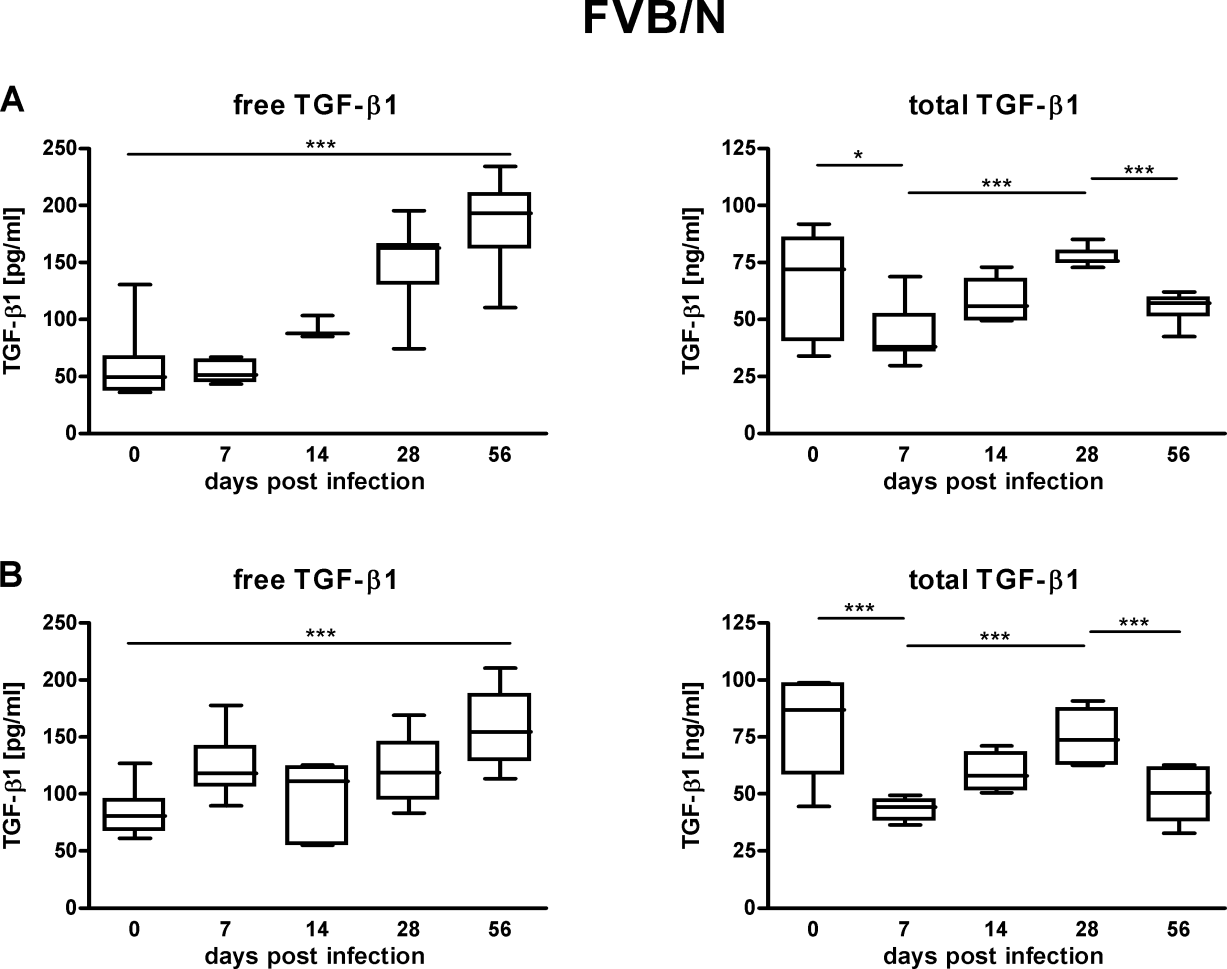
TGF-*β*1 content in the sera of FVB/N mice suffering from lyme disease. Measured concentrations of free (pg/ml) as well as total (ng/ml) TGF-*β*1 in the sera of FVB/N wild type mice 0, 7, 14, 28 and 56 days post infection with *B. burgdorferi sensu stricto* N40. Data are from 2 independent experiments with 7 animals tested at each time point (*N* = 2, *n* = 7). (A) Different mice were sacrificed at every time point to take cardiac blood samples. (B) The same mice were bled retro-orbitally multiple times during the course of the infection. Statistical analysis was carried out for each experiment using the program GraphPad Prism 4 (GraphPad Software, La Jolla, USA). One-way analysis of variance followed by unpaired Students *t* test was used to identify significant differences and correlations between means (* *p* < 0.05, *** *p* < 0.001).

In blood serum TGF-*β*1 occurs in two types: the latent precursor and the active cytokine. To obtain an overall picture both the active TGF-*β*1 content and the total TGF-*β*1 content, that is the concentration of active plus latent TGF-*β*1, were evaluated. In the course of lyme disease for both mouse strains tested there was an increase in the serum content of active TGF-*β*1. Compared to uninfected mice the increase in active TGF-*β*1 serum content became significant 56 days post infection (*p* < 0.001) (Figs. 1 and 2). The concentration profile measured parallels the situation seen for hepatitis c and other infectious diseases (Barral et al., 1993; Cecere et al., 2004; Dahl et al., 1996; Reinhold et al., 1999; Schultz-Cherry & Hinshaw, 1996; Toossi et al., 1995). The marked rise of active TGF-*β*1 content as a result of *Borrelia* infection underlines that the cytokine, in its active form, meets a critical prerequisite to be used as a prognostic marker in lyme disease. However, the restriction must be made that partially there are considerable individual variations in the active TGF-*β*1 concentrations determined (Figs. 1 and 2). For both mouse strains tested the serum concentration of total TGF-*β*1 in the course of infection initially decreased then rebounded and subsequently dropped again (Figs. 1 and 2). This alternating concentration profile renders the total TGF-*β*1 content unsuitable as a diagnostic tool. There were no differences in TGF-*β*1 serum profiles from mice bled cardiac one times or retro-orbital multiple times.

### Relationship of serum content of active TGF-*β*1 to lyme arthritis

**Figure 3:**
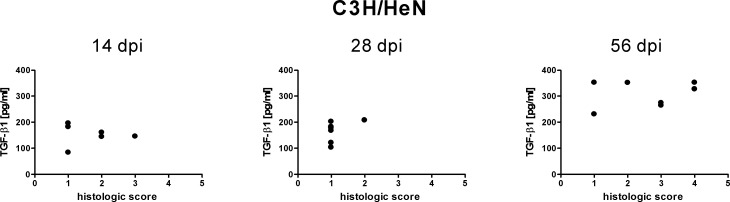
Relationship of free TGF-*β*1 content in the sera of C3H/HeN mice to inflammatory lessions. The measured concentrations of free TGF-*β*1 in the sera of C3H/HeN wild type mice 14, 28 and 56 days post infection (dpi) with *B. burgdorferi sensu stricto* N40 were plotted against the histologic score of the tibiotarsal joints of those mice at these time points. The histologic score used ranged from 1 to 6; score 1 denoted no pathologic changes, score 6 denoted high-grade changes. Results of the linear regression analysis: 14 dpi: *R*^2^ = 0.006351, *p* = 0.8807; 28 dpi: *R*^2^ = 0.2177, *p* = 0.2913; 56 dpi: *R*^2^ = 0.04766, *p* = 0.6381. Data are from 1 independent experiment with 7 animals tested at each time point (*N* = 1, *n* = 7). Statistical analysis was carried out using the program GraphPad Prism 4 (GraphPad Software, La Jolla, USA).

**Figure 4:**
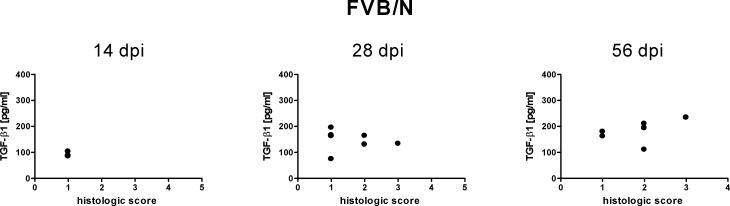
Relationship of free TGF-*β*1 content in the sera of FVB/N mice to inflammatory lessions. The measured concentrations of free TGF-*β*1 in the sera of FVB/N wild type mice 14, 28 and 56 days post infection (dpi) with *B. burgdorferi sensu stricto* N40 were plotted against the histologic score of the tibiotarsal joints of those mice at these time points. The histologic score used ranged from 1 to 6; score 1 denoted no pathologic changes, score 6 denoted high-grade changes. Results of the linear regression analysis: 14 dpi: *R*^2^ = 0.003119, *p* = 0.9163; 28 dpi: *R*^2^ = 0.01917, *p* = 0.7672; 56 dpi: *R*^2^ = 0.2176, *p* = 0.2913. Data are from 1 independent experiment with 3 animals tested at time point 14 dpi and 7 animals tested at time points 28 dpi and 56 dpi. Statistical analysis was carried out using the program GraphPad Prism 4 (GraphPad Software, LaJolla, USA).

Despite considerable individual variations in active TGF-*β*1 serum concentrations there were no identifiable dissimilarities in the clinical appearance of the mice. Likewise, for both mouse strains tested no correlation could be seen between the serum content of active TGF-*β*1 and the severity of lyme arthritis of tibiotarsal joints of infected mice (Figs. 3 and 4). The histologic score, which symbolizes the extent of inflammatory lesions, did not increase with increasing active TGF-*β*1 and vice versa (Figs. 3 and 4). It should be noted that mice with an almost identical serum level of active TGF-*β*1 exhibited highly variable histological scores (Figs. 3 and 4). This becomes particularly obvious 56 days post infection, a time point when lyme arthritis is clearly defined (Figs. 3 and 4).

### Validity of the findings

The experiments were performed using two different wild type mouse strains: C3H/HeN and FVB/N. The data collected for the two wild type strains paralleled each other. Both for C3H/HeN mice as well as for FVB/N mice *Borrelia* infection was found to go along with a significant increase in active TGF-*β*1 content and an alternating concentration profile of total TGF-*β*1. In addition, both for C3H/HeN mice as well as for FVB/N mice there was no correlation between the serum content of active TGF-*β*1 to lyme arthritis. Moreover, in reference to the data the blood collection technique (cardiac one times versus retro-orbital multiple times) had no influence on the TGF-*β*1 serum content measured.

## Conclusions

The present study clearly shows that TGF-*β*1 is of no avail as a prognostic marker in lyme disease. As described for numerous inflammatory conditions (Barral et al., 1993; Cecere et al., 2004; Dahl et al., 1996; Reinhold et al., 1999; Schultz-Cherry & Hinshaw, 1996; Toossi et al., 1995) *Borrelia* infections goes along with a significant increase in active TGF-*β*1 serum content. However, there is no correlation between the level of active TGF-*β*1 concentration in blood serum and disease’s clinical severity. Hence, the search for an endogenous predictive factor, which can be determined in an easy and reliable manner, remains open.

## References

[ref-1] Barral A, Barral-Netto M, Yong EC, Brownell CE, Twardzik DR, Reed SG (1993). Transforming growth factor beta as a virulence mechanism for Leishmania braziliensis. Proceedings of the National Academy of Sciences of the United States of America.

[ref-2] Bettelli E, Carrier YJ, Gao WD, Korn T, Strom TB, Oukka M, Weiner HL, Kuchroo VK (2006). Reciprocal developmental pathways for the generation of pathogenic effector T(H)17 and regulatory T cells. Nature.

[ref-3] Burchill MA, Nardelli DT, England DM, DeCoster DJ, Christopherson JA, Callister SM, Schell RF (2003). Inhibition of interleukin-17 prevents the development of arthritis in vaccinated mice challenged with Borrelia burgdorferi. Infection and Immunity.

[ref-4] Cecere A, Marotta F, Vangieri B, Tancredi L, Gattoni A (2004). Progressive liver injury in chronic hepatitis C infection is related to altered cellular immune response and to different citokine profile. Panminerva Medica.

[ref-5] Dahl KE, Shiratsuchi H, Hamilton BD, Ellner JJ, Toossi Z (1996). Selective induction of transforming growth factor beta in human monocytes by lipoarabinomannan of Mycobacterium tuberculosis. Infection and Immunity.

[ref-6] Fortunel NO, Hatzfeld A, Hatzfeld JA (2000). Transforming growth factor-beta: pleiotropic role in the regulation of hematopoiesis. Blood.

[ref-7] Knauer J, Siegemund S, Muller U, Al-Robaiy S, Kastelein RA, Alber G, Straubinger RK (2007). Borrelia burgdorferi potently activates bone marrow-derived conventional dendritic cells for production of IL-23 required for IL-17 release by T cells. FEMS Immunology and Medical Microbiology.

[ref-8] Nardelli DT, Burchill MA, England DM, Torrealba J, Callister SM, Schell RF (2004). Association of CD4(+) CD25(+) T cells with prevention of severe destructive arthritis in Borrelia burgdorferi-vaccinated and challenged gamma interferon-deficient mice treated with anti-interleukin-17 antibody. Clinical and Diagnostic Laboratory Immunology.

[ref-9] Nardelli DT, Luk KHK, Kotloski NJ, Warner TF, Torrealba JR, Callister SM, Schell RF (2008). Role of IL-17, transforming growth factor-beta, and IL-6 in the development of arthritis and production of anti-outer surface protein A borreliacidal antibodies in Borrelia-vaccinated and -challenged mice. FEMS Immunology and Medical Microbiology.

[ref-10] Reinhold D, Wrenger S, Kahne T, Ansorge S (1999). HIV-1 Tat: immunosuppression via TGF-beta1 induction. Immunology Today.

[ref-11] Schultz-Cherry S, Hinshaw VS (1996). Influenza virus neuraminidase activates latent transforming growth factor beta. Journal of Virology.

[ref-12] Schumann J, Blessing M (2011). The Impact of TGF-beta, GM-CSF and antibody response for diagnosis as well as etiopathology of lyme disease. Scandinavian Journal of Laboratory Animal Science.

[ref-13] Steere AC (2001). Medical progress: lyme disease. New England Journal of Medicine.

[ref-14] Straubinger RK, Straubinger AF, Summers BA, Jacobson RH (2000). Status of Borrelia burgdorferi infection after antibiotic treatment and the effects of corticosteroids: an experimental study. Journal of Infectious Diseases.

[ref-15] Toossi Z, Young TG, Averill LE, Hamilton BD, Shiratsuchi H, Ellner JJ (1995). Induction of transforming growth factor beta 1 by purified protein derivative of Mycobacterium tuberculosis. Infection and Immunity.

[ref-16] Veldhoen M, Hocking RJ, Atkins CJ, Locksley RM, Stockinger B (2006). TGF beta in the context of an inflammatory cytokine milieu supports de novo differentiation of IL-17-producing T cells. Immunity.

[ref-17] Wahl SM (1992). Transforming growth-factor-beta (Tgf-Beta) in inflammation—a cause and a cure. Journal of Clinical Immunology.

[ref-18] Wahl SM (1994). Transforming growth-factor-beta—the good, the bad, and the ugly. Journal of Experimental Medicine.

[ref-19] Wahl SM (1999). TGF-beta in the evolution and resolution of inflammatory and immune processes—introduction. Microbes and Infection.

